# Botulinum Toxin Type A as an Early Intervention for Traumatic Oculomotor Nerve Palsy: A Pediatric Case Report

**DOI:** 10.7759/cureus.105092

**Published:** 2026-03-12

**Authors:** Chihiro Koiwa, Takashi Negishi, Megumi Ito, Eiichiro Noda, Shintaro Nakao

**Affiliations:** 1 Department of Ophthalmology, Faculty of Medicine, Juntendo University, Tokyo, JPN; 2 Department of Ophthalmology, Juntendo University, Tokyo, JPN; 3 Department of Ophthalmology, School of Medicine, Juntendo University, Tokyo, JPN; 4 Department of Ophthalmology, Tokyo Metropolitan Children’s Medical Center, Tokyo, JPN

**Keywords:** botulinum toxin type a, oculomotor nerve injury, pediatric head trauma, post-traumatic strabismus, traumatic third cranial nerve palsy

## Abstract

Traumatic third cranial nerve palsy is a rare complication of head injury, with an incidence of approximately 1% and a characteristically poor prognosis. Conventional management remains conservative, often yielding unsatisfactory outcomes. We report the case of a 13-year-old girl who developed complete right third cranial nerve palsy following a 15-meter fall, presenting with exotropia (35Δ), ptosis, complete ophthalmoplegia, and pupillary dysfunction. Brain CT revealed hemorrhage in the right cavernous sinus, and subsequent MRI demonstrated focal nerve damage. Thirty-eight days post-injury, a single botulinum toxin type A (BTX-A) injection (5 units) was administered to the right lateral rectus muscle. Progressive improvement in ocular alignment and motility was observed, with resolution of diplopia by 4.5 months and sustained orthotropia at 10 months post-injury. Although pupillary dilation persisted, functional recovery was substantial. This case demonstrates that early BTX-A intervention may prevent lateral rectus contracture and promote functional recovery in traumatic third cranial nerve palsy. BTX-A represents a promising minimally invasive therapeutic option that warrants further investigation in larger patient populations.

## Introduction

Among cranial nerve palsies caused by head injuries, primary oculomotor nerve (third cranial nerve) palsy, excluding secondary complications caused by brain herniation, accounts for approximately 1% of cases and is relatively rare [1-3]. The third cranial nerve controls several extraocular muscles responsible for eyelid elevation and pupil constriction [4]. Therefore, injury to this nerve can result in symptoms such as ptosis, ophthalmoplegia, and pupillary dysfunction, which can significantly impair a patient's visual function and quality of life [4-6]. The clinical course of traumatic third cranial nerve palsy is often unpredictable. While some patients experience spontaneous recovery, others may suffer from persistent deficits such as diplopia and strabismus, for which effective therapeutic interventions are limited [7,8].

The conventional approach to managing traumatic third cranial nerve palsy is often conservative, involving observation and supportive measures during the acute phase [9]. However, these approaches may be insufficient, particularly given the low recovery rate [9]. In recent years, botulinum toxin type A (BTX-A) has emerged as a minimally invasive treatment option for various ocular motility disorders, including strabismus secondary to nerve palsy [10]. BTX-A acts by temporarily weakening overacting extraocular muscles, thereby improving ocular alignment and reducing symptoms such as diplopia [10].

Despite its established role in other forms of strabismus, the use of BTX-A in traumatic third cranial nerve palsy remains relatively underreported, and its efficacy in this context is not well understood. Here, we present a case of primary third cranial nerve palsy following head trauma that was successfully managed with BTX-A therapy. This case highlights the potential benefits of BTX-A as an early intervention for traumatic third cranial nerve palsy and contributes to the growing body of evidence supporting its use in neuro-ophthalmological disorders.

## Case presentation

A 13-year-old girl was emergently transported to a pediatric trauma center following a 15-meter fall from a pedestrian overpass, resulting in multiple traumatic injuries, including craniofacial fractures, pulmonary contusion, lumbar spine fracture, and bilateral lower extremity fractures. On initial evaluation, anisocoria (right pupil: 5 mm, left pupil: 2 mm), right-sided ptosis, and impaired extraocular movements were observed. Brain CT revealed hemorrhage in the right cavernous sinus. Due to the need for stabilization of her general condition, ophthalmologic evaluation was delayed until one month after injury.

**Figure 1:**
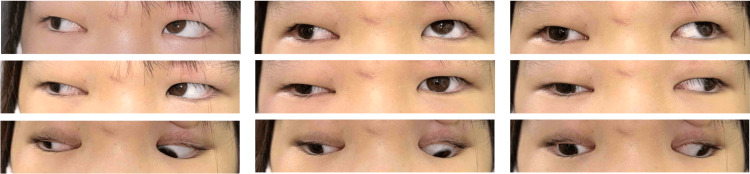
Nine-gaze eye photo before botulinum toxin injection Exotropia is observed in the primary position. Ptosis and ocular motility disorder in the right eye, except for abduction, are also noted.

At initial ophthalmologic assessment, the patient demonstrated complete right third cranial nerve palsy characterized by marked limitation of adduction (-4), elevation (-3), and depression (-3), accompanied by right exotropia (Krimsky test: 35Δ XT at near and 25Δ XT at distance), ptosis, and a dilated, non-reactive right pupil (Figure 1). Visual acuity was 0.1 (0.3 × S -0.50D C -1.50D A 180°) in the right eye and 0.3 (1.0 × S -1.25D) in the left eye. No abnormalities were noted in the anterior or posterior segments bilaterally except for fixed mydriasis in the right eye.

**Figure 2:**
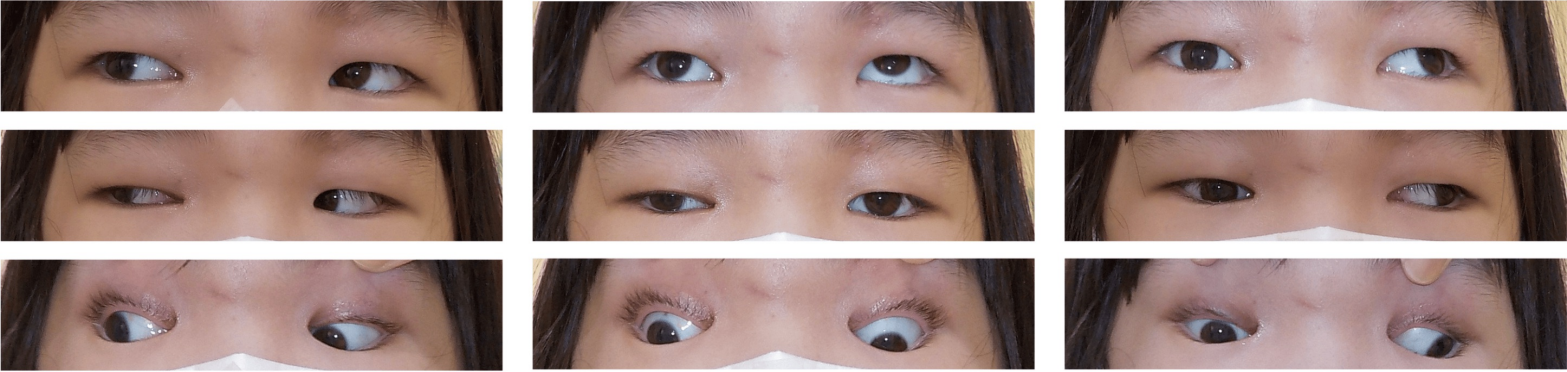
Nine-gaze eye photo on the eighth day after botulinum toxin injection Adduction across the midline was observed.

**Figure 3:**
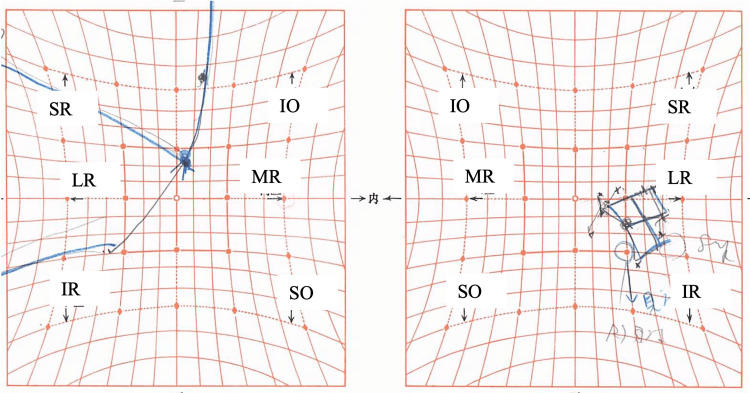
Hess chart on the eighth day after botulinum toxin injection Ocular motility disorder in the right eye is observed, with corresponding overaction of the left eye. MR, medial rectus; LR, lateral rectus; SR, superior rectus; IR, inferior rectus; SO, superior oblique; IO, inferior oblique

Thirty-eight days after the injury, a single injection of BTX-A (5 units in 0.05 mL) was administered into the right lateral rectus muscle. Eight days post-injection, ocular alignment improved with a reduction of right exotropia to 18Δ XT' at near and 18Δ XT at distance, and mild improvement in adduction was noted (Figure 2 and Figure 3).

**Figure 4:**
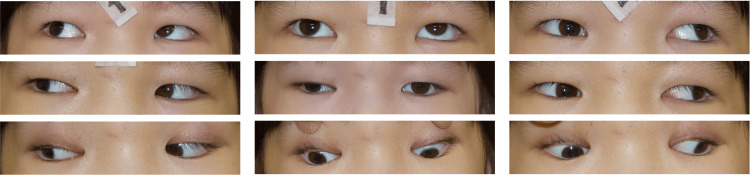
Nine-gaze eye photo three weeks after botulinum toxin injection Although right ptosis remains, ocular motility has improved compared to before the botulinum toxin injection.

**Figure 5:**
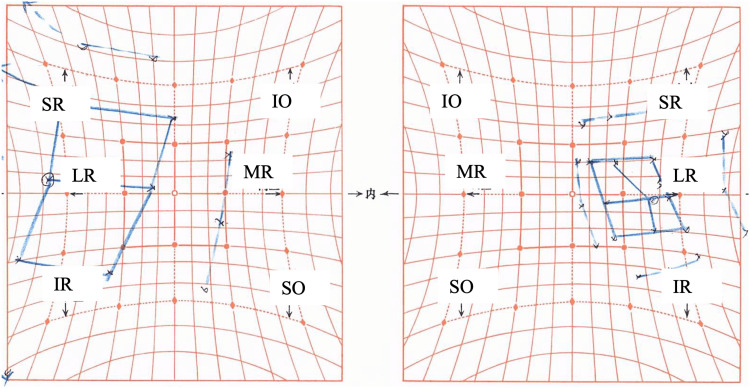
Hess chart three weeks after botulinum toxin injection A slight improvement in ocular motility of the right eye is observed over time. MR, medial rectus; LR, lateral rectus; SR, superior rectus; IR, inferior rectus; SO, superior oblique; IO, inferior oblique

At three weeks post-injection (two months after injury), further improvement in adduction allowed the right eye to cross the midline (Figure 4 and Figure 5).

**Figure 6:**
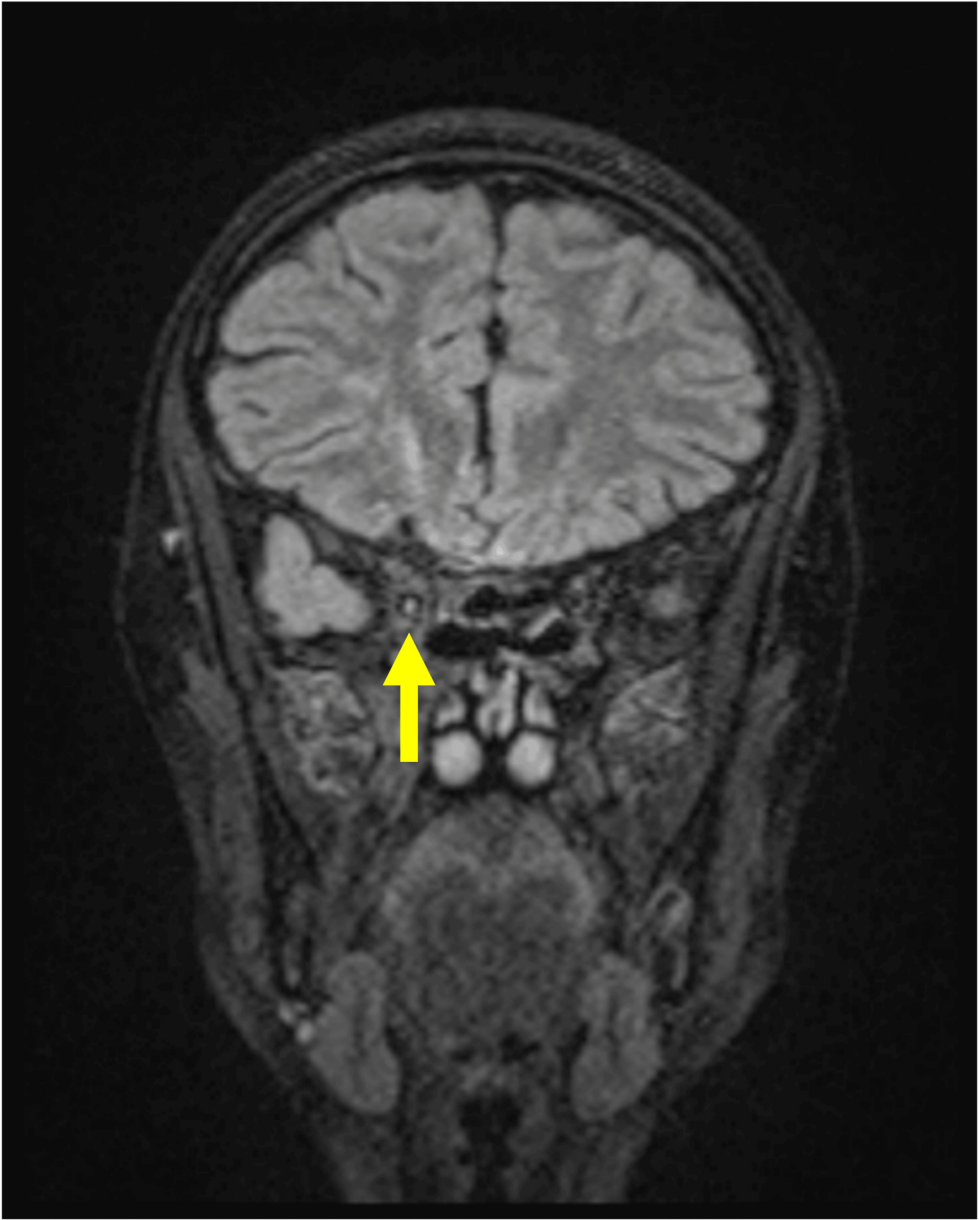
Head MRI performed approximately two months after injury A localized area of increased signal intensity suggestive of damage to the right oculomotor nerve was observed on fat-suppressed 3D-FLAIR images (yellow arrow). FLAIR, fluid-attenuated inversion recovery

Titmus stereo testing showed improved stereopsis (Fly (+), Animals 3/3, Circles 7/9). Also, an MRI performed approximately two months post-injury revealed a focal high-signal lesion in the right third cranial nerve on fat-suppressed 3D-fluid-attenuated inversion recovery (FLAIR) images, consistent with localized nerve damage (Figure 6). No other structural brain abnormalities were noted.

**Figure 7:**
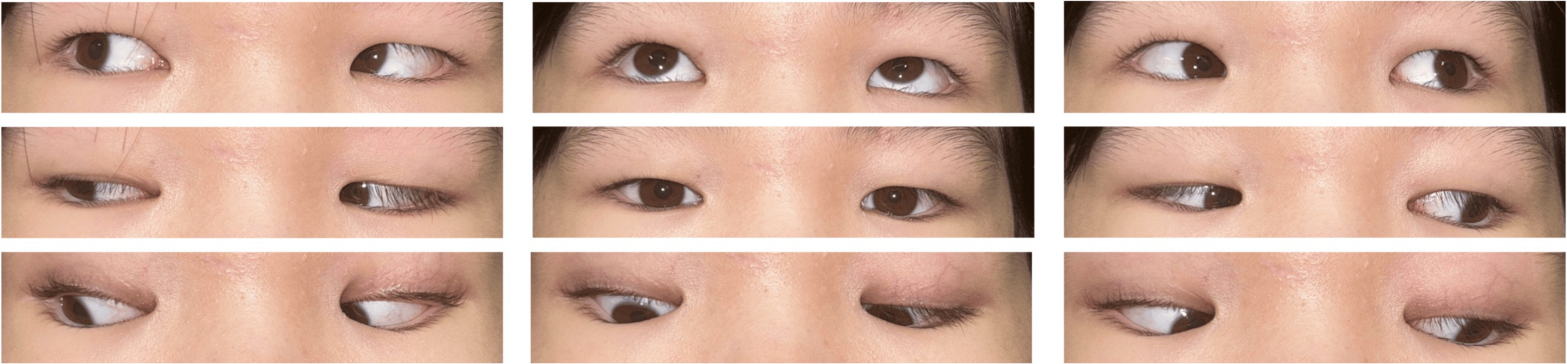
Nine-gaze eye photo three months after botulinum toxin injection Orthotropia in the primary position has been achieved. Ptosis has resolved. Mild right mydriasis is present.

**Figure 8:**
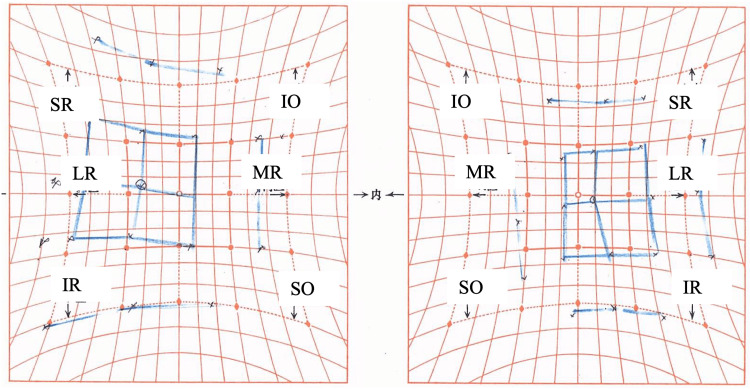
Hess chart three months after botulinum toxin injection Ocular motility continues to improve, although limitation in adduction persists. MR, medial rectus; LR, lateral rectus; SR, superior rectus; IR, inferior rectus; SO, superior oblique; IO, inferior oblique

By three months post-injection (4.5 months after injury), ocular alignment improved to near 8Δ XT' and orthotropia at distance, with complete resolution of subjective diplopia (Figure 7 and Figure 8).

**Figure 9:**
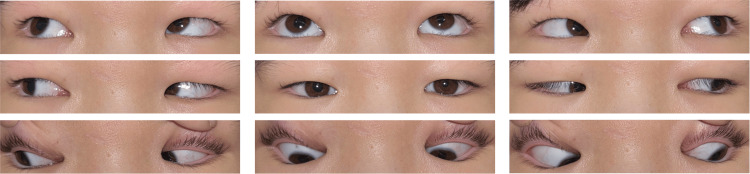
Nine-gaze eye photo 5.5 months after botulinum toxin injection Ocular motility disorder has almost completely resolved.

**Figure 10:**
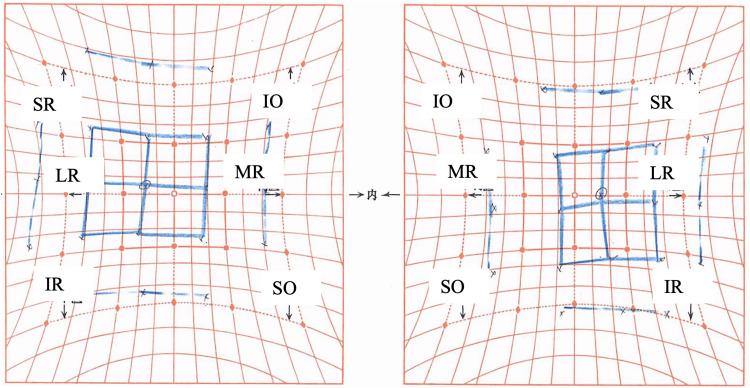
Hess chart 5.5 months after botulinum toxin injection A slight limitation in right eye adduction is observed. MR, medial rectus; LR, lateral rectus; SR, superior rectus; IR, inferior rectus; SO, superior oblique; IO, inferior oblique

Visual acuity improved to 0.6 in the right eye and 0.2 in the left eye. At 5.5 months post-injection (seven months after injury), the patient maintained orthotropia at both near and distance (Figure 9 and Figure 10).

**Figure 11:**
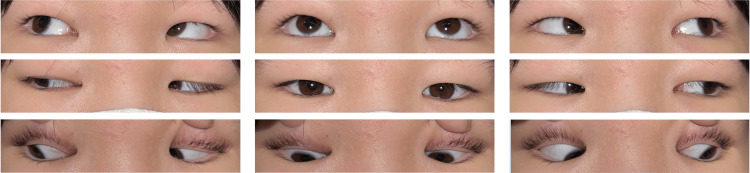
Nine-gaze eye photo 8.5 months after botulinum toxin injection Ocular motility disorder improved. Pupillary diameter enlargement of the right eye remained, but there was no photophobia.

**Figure 12:**
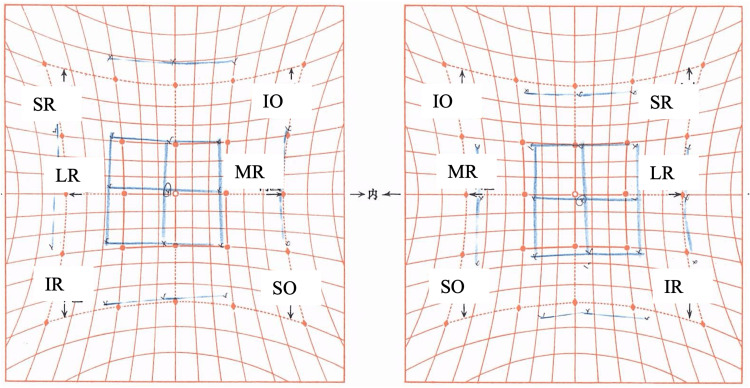
Hess chart 8.5 months after botulinum toxin injection Ocular motility disorder in the right eye has resolved. MR, medial rectus; LR, lateral rectus; SR, superior rectus; IR, inferior rectus; SO, superior oblique; IO, inferior oblique

By 8.5 months post-injection (10 months after injury), the patient exhibited sustained ocular alignment and motility improvement, with full adduction and no residual diplopia. Although right pupillary dilation persisted, the patient reported no subjective photophobia (Figure 11 and Figure 12).

## Discussion

Traumatic third cranial nerve palsy is a rare complication of head injury, with reported incidence rates ranging from 1.1% to 1.2% among patients with head trauma [1-3]. Previous studies have emphasized not only the low incidence of this condition but also its poor prognosis. The recovery rate for traumatic third cranial nerve palsy is significantly lower than for nontraumatic causes such as ischemia [3,11]. In fact, previous studies of traumatic cases have shown that although ptosis recovers in up to 78% of cases, extraocular muscle paralysis improves in 44% of cases, and pupillary dysfunction recovers in only 20% [12].

Conventional management for traumatic third cranial nerve palsy is often conservative, with observation and supportive measures in the acute phase [9]. However, these approaches may not be sufficient, especially given the low recovery rate. BTX-A therapy has emerged as a promising, minimally invasive option for the management of ocular motility disorders, including those caused by cranial nerve palsies [10]. In patients with traumatic or vascular third cranial nerve palsy, BTX-A injections into the lateral rectus muscle have been reported to improve eye alignment and reduce diplopia [10]. A meta-analysis of 38 studies on BTX-A therapy found that the treatment success rate was 79% in acute cases of abducens nerve palsy (sixth cranial nerve), compared to 33% in chronic cases. For acute cases, BTX-A therapy was concluded to yield significantly better results than observation alone [10]. Talebnejad et al. reported the use of BTX-A injections into the ipsilateral lateral rectus muscle in nine patients with acute traumatic partial third cranial nerve palsy presenting with exotropia within two months of onset [13]. Following treatment, the mean deviation decreased from 48.3Δ exotropia before injection to 14.2Δ at final follow-up. Seven of nine patients (77.8%) achieved single binocular vision in the primary position, while two patients subsequently required strabismus surgery. These findings suggest that early BTX-A intervention may be effective in restoring binocular function in acute traumatic third cranial nerve palsy.

In the present case, a single BTX-A injection led to sustained improvement in ocular motility and subjective diplopia for up to eight months. While some degree of spontaneous recovery cannot be excluded, the low natural recovery rate in traumatic third cranial nerve palsy suggests that early intervention with BTX-A may have played a significant role in preventing contracture of the lateral rectus muscle and promoting functional recovery. This case supports previous findings that BTX-A therapy can be an effective early treatment option for traumatic third cranial nerve palsy, potentially improving outcomes and reducing the need for more invasive interventions.

This case report should be considered hypothesis-generating and descriptive in nature, and caution should be exercised in drawing definitive conclusions from a single case. Further studies with larger patient populations and longer follow-up are warranted to better define the optimal timing, dosing, and patient selection for BTX-A therapy in this context. Nonetheless, this case adds to the growing body of evidence supporting BTX-A as a valuable therapeutic tool in the management of traumatic third cranial nerve palsy. Although reports of significant efficacy are limited, the favorable outcome is thought to have resulted from the patient's young age and early intervention.

## Conclusions

In conclusion, in this case, sustained improvement in ocular motor function and a subjective reduction in diplopia were observed for up to eight months following a single administration of BTX-A. While the possibility of spontaneous recovery cannot be ruled out, considering the low rate of spontaneous recovery in traumatic third cranial nerve palsy, early intervention with BTX-A may play an important role in preventing muscle spasticity of the lateral rectus muscle and promoting functional recovery. This case supports the potential of BTX-A therapy as an effective early treatment option not only for sixth cranial nerve palsy but also for traumatic third cranial nerve palsy. However, it should be emphasized that these findings provide support rather than conclusive evidence, and further prospective studies are needed to confirm the efficacy of BTX-A in this setting.
